# Synthesis, Properties, and Applications of Morphology-Controlled Perovskite Quantum Dots

**DOI:** 10.1021/acsaom.5c00607

**Published:** 2026-02-09

**Authors:** Matthew L. Atteberry, Chenjia Mi, Sohom Chandra, Sania Fiaz, Yitong Dong

**Affiliations:** † Department of Chemistry and Biochemistry, 6187 The University of Oklahoma, Norman, Oklahoma 73019, United States; ‡ Center for Quantum Research and Technology, The University of Oklahoma, Norman, Oklahoma 73019, United States

**Keywords:** perovskite nanocrystals, morphology, surface ligands, exciton, photocatalysis

## Abstract

Lead halide perovskite quantum dots (QDs) have become a promising class of nanomaterials due to their simple, scalable synthesis and high luminescence efficiency. However, their high ionicity and low lattice formation energy make controlling the synthesis of perovskite QDs particularly challenging. Although there have been significant advances in controlling the size of perovskite QDs, increasing efforts have focused on selecting and stabilizing their various surface facets. In this review, we examine recent developments in morphology-controlled isotropic perovskite QDs, emphasizing the latest techniques for managing surface facet exposure, facet passivation, and the optical and chemical properties of these QDs. We also explore future challenges and opportunities for precise synthesis control, especially regarding shape control of strongly confined QDs, which is vital for understanding the relationship between structure and properties—ultimately improving the performance and stability of perovskite QD-based optoelectronic devices and photocatalysts.

## Graphical abstract

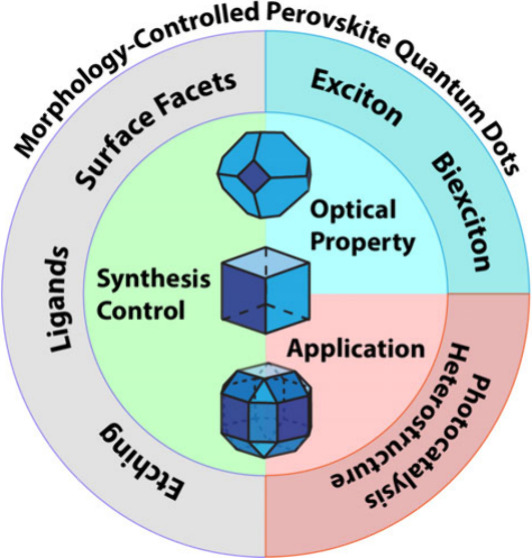

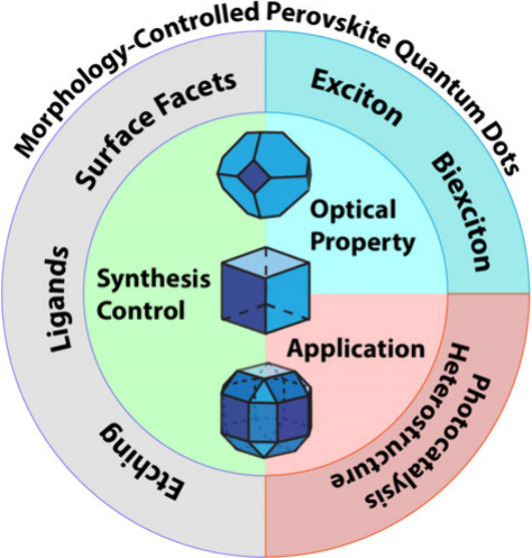

## 1 Introduction

Colloidal nanocrystal quantum dots (QDs) have a high surface-to-volume ratio, making their physical and chemical properties highly dependent on their surfaces.^1^ For example, atoms on the surface chemically bind to ligands, thereby improving colloidal stability.^2^ Surface defects can trap charge carriers, significantly affecting the electronic and optical properties of QDs.^3,4^ Moreover, the chemical properties of exposed surface facets can influence photocatalytic performance.^5–7^ Although QDs are generically considered nanospheres, their actual morphology is typically polyhedral, with various facets exposed. Therefore, the morphology control of QDs is vital for tuning optical properties, forming ordered self-assemblies, and designing new photocatalysts.^8–10^ To date, synthesis control of the QD shape and studies on the surface facet–property relationships have been applied to II–VI,^11–13^ III–V,^14–16^ and IV–VI^17^ QDs.

Recently, colloidal lead halide perovskite (LHP) QDs following the stoichiometry of APbX_3_ (A = Cs^+^, FA^+^, MA^+^; X = Cl, Br, I) have gained significant attention for their exceptional photophysical properties, including their inherent defect tolerance and fast radiative rates at cryogenic temperatures.^18–20^ This unique combination makes LHP QDs well-suited for highly efficient light-emitting diodes and bright quantum light sources: state-of-the-art LHP QD LEDs have achieved an external quantum efficiency of >20%.^21–24^ Recently, single-photon emission from LHP QDs with high brightness and stability has been demonstrated at room temperature.^25,26^ At cryogenic temperatures, CsPbBr_3_ nanocrystals have also demonstrated high photon indistinguishability and superradiance.^27,28^ Aside from their remarkable optical properties, one of the main advantages of LHP QDs is their facile synthesis and flexible compositional tunability: compared to other nanomaterials and traditional colloidal QDs, the synthesis of LHP QDs is straightforward and highly adaptable, with rapid anion and A-site cation exchange enabling simple tuning of composition and bandgap, making them ideal for color-tunable light sources and photovoltaics.^29^ Due to these features, LHP QDs have also been used in photodetectors, scintillators, and chemical sensors.^30–36^

The highly ionic LHP lattices have low formation energy and high ion mobility. While this facilitates compositional adjustment of LHP QDs, it also poses challenges for regulating their size and surface morphology. The rapid growth rate, soft lattice structure, and dynamic ligand binding of LHP QDs make them vulnerable to increased inhomogeneities in size and surface features, as well as limited structural stability.^37,38^ Since the initial demonstrations of hybrid and inorganic LHP,^18,39^ significant progress has been made in understanding the formation and reaction mechanisms during LHP QD solution-based syntheses with precise size control.^40–44^ However, most reported colloidal synthesis methods produce cube-shaped LHP QDs mainly exposing the (100) facet of the cubic phase or the equivalent (110) facet of the orthorhombic phase.^45,46^ While multifaceted QDs have been reported, controlling their surface morphology is often inadequate or unintentional. To date, achieving precise control over surface facets in LHP QDs remains an open challenge.

Facet engineering of bulk LHP crystals has recently been shown to be essential for achieving stable and efficient photovoltaic performance.^47^ In cubic LHP crystals, (100) facets are considered to have balanced coordination, with the A-site cations and halide anions neighboring each other. Such an AX surface termination has been proven to ensure charge neutrality and be free of electronic defects.^48^ In comparison, the (111) facet of the LHP contains more halide anions than A-site cations, leading to an overall negative charge. Similarly, the (110) facets display unbalanced charge but are instead positively charged. Figure 1 illustrates the atomic structure of these facets. The different atomic compositions of the facets will result in distinct thermodynamic and kinetic properties for their growth and dissolution. Furthermore, the varying degrees of surface coordination unsaturation will create different defect energy levels on each facet, influencing their optical and chemical properties. For colloidal LHP QDs, surface ligands and their interactions with various facets also play a key role in facet characteristics. Despite the importance of surface facet exposure, to our knowledge, few reviews and discussions have addressed the relationship between the surface morphology and the properties of LHP QDs.

**Figure 1:**
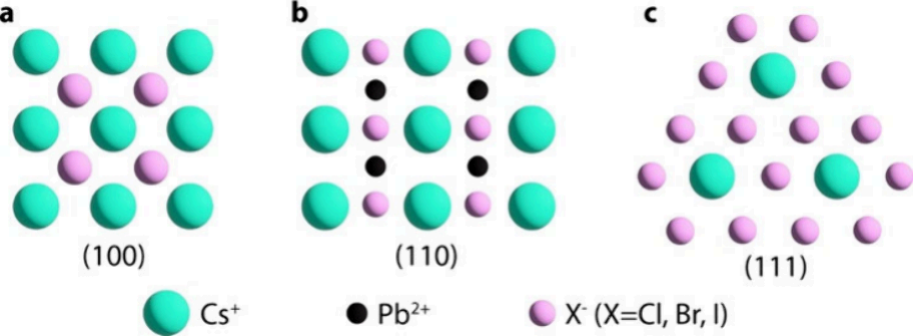
Atomic arrangements of perovskite crystal facets on an ideal cubic CsPbX_3_ crystal: (a) (100); (b) (110); (c) (111).

In this review, we examine recent contributions on the synthetic parameters that control facet exposure in LHP QDs, discuss their impact on optical properties, and applications such as quantum light sources and photocatalytic reactions. We detail the chemical identities of each facet on LHP QDs and explore current advances in promoting the growth and formation of different facets. We review recent theoretical and experimental studies demonstrating how facet- or surface-morphology-dependent optical properties manifest. Finally, we discuss the influence of QD surface facets or morphologies on the performance of LHP QD lasing/amplified spontaneous emission (ASE) and photocatalytic activities. Extending our understanding of detailed structural-property relationships remains a key priority for the future development of LHP nanocrystals.

## 2 Synthesis of LHP QDs with Different Morphologies

Controlling the shape of LHP QDs is crucial for understanding how surface facets affect their photophysical properties. To accurately relate spectroscopic data to QD features, high ensemble uniformity is essential. As described in the Introduction, the rapid growth of LHP QDs, driven by their soft lattice and dynamic surface ligand binding, makes stabilizing surface facets more difficult compared to traditional II–VI QDs. Most LHP QDs are cuboidal and primarily expose (100) facets. It remains unclear how the stability of different facets and their formation kinetics interact during synthesis. Additionally, stabilizing facets other than (100) is still in early stages, and the evolution of facets during synthesis and purification can influence the final surface shape of LHP QDs. This section reviews strategies to direct the growth and stabilization of different surface facets.

### 2.1 Solvent-Assisted Chemical Etching and Surface Reconstruction

Surface ligand passivation can stabilize exposed surface facets. It is widely accepted that the binding of many ligands on LHP QDs is relatively weak and dynamic.^49,50^ As a result, certain chemicals can remove surface ligands, thereby destabilizing or even dissolving the facets. Additionally, ligand desorption and QD morphology change can also occur when QDs are excited.^51,52^ This phenomenon was observed during the purification of QDs using polar antisolvents, leading to shape transformation. A common method involves adding large amounts of polar antisolvents to precipitate LHP QDs. Ye et al. demonstrated that the polarity of the antisolvent significantly influences the structural integrity of CsPbBr_*x*_I_3–*x*_ QDs (Figure 2b).^53^ Highly polar antisolvents such as acetone and 1-butanol promote ligand desorption via amide condensation reactions and, subsequently, induce surface iodide anion loss. Mei et al. also observed that, to colloidal LHP QDs, adding polar solvents, such as ethanol and acetone can cause disimilar mophologies and size reduction (Figure 2c–e) due to ligand desorption and surface etching, whereas adding nonpolar solvents, such as toluene and hexane, helps to maintain the cubic shape of CsPbBr_3_ QDs by minimizing surface ligand removal.^54^ Chiba et al. have further demonstrated this, showing that alcohols such as butanol can promote undesirable growth of QDs during washing.^55^ Li et al. have demonstrated that excessive antisolvent reprecipitation cycles of CsPbBr_3_ QDs result in irregular particle morphologies and uncontrolled growth due to ligand loss.^56^ In a study by Sun et al., it is demonstrated that polar solvents, such as dimethylformamide (DMF), can remove a large number of ligands from LHP QDs, leading to coalescence (Figure 2f–h).^57^ Additionally, tetramethylethylenediamine will partially replace the long-chain organic ligands on LHP QDs and result in QD morphology changes. The photoinduced ligand detaching and formation of Pb^0^ have been investigated by An et al. (Figure 2i–k), and the photodegraded QDs exhibit a transfer from cubical to spherical shapes. Ligand detachment under photoexcitation has also been observed alongside aggregation for CsPbBr_3_ QDs.^52,53^

**Figure 2:**
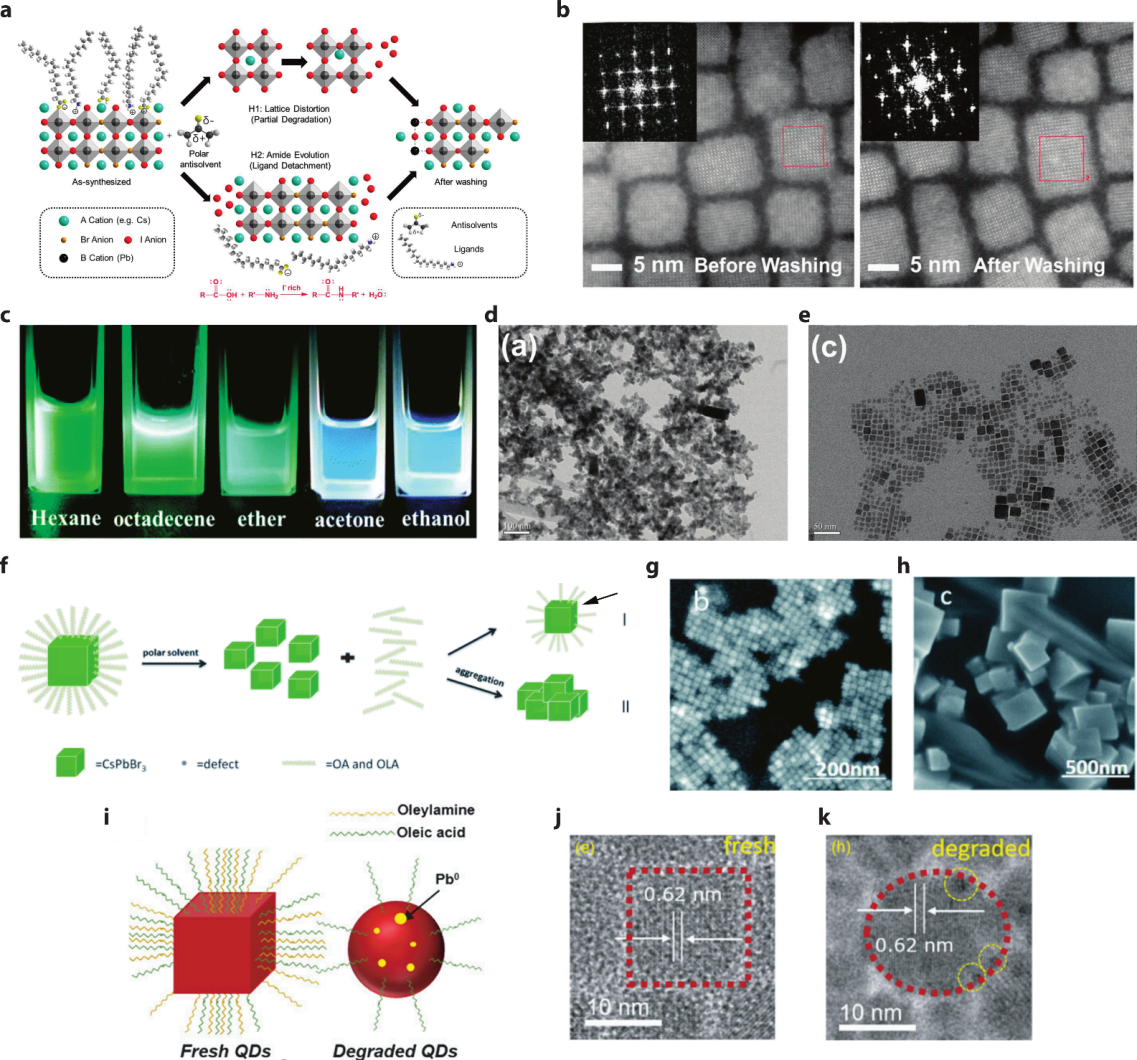
(a) Schematic illustration of the proposed mechanisms for the antisolvent-dependent selective etching of the mixed I/Br perovskite QDs. First proposed route (H1): solvent-induced lattice distortion, which results in nanocrystal degradation and the formation of PbI_2_. Second proposed route (H2): the addition of a polar solvent, which results in amide formation and leads to a surface ligand and iodide detachment. (b) STEM of the as-synthesized (left) and CsPbBr_*x*_I_3–*x*_ nanocrystals washed with acetone (right). The inset in the STEM images contains the fast Fourier transform images of the nanocrystals in the selected red region of the images. Panels a and b are reproduced with permission from ref 53. Copyright 2022 American Chemical Society. (c) Fluorescence images of CsPbBr_3_ nanocrystals suspended in polar and nonpolar solvents (hexane, octadecene, ether, acetone, and ethanol) under UV excitation. (d and e) TEM images of CsPbBr_3_ QDs dissolved in polar ethanol (d) and nonpolar hexane (e). Panels c–e are reproduced with permission from ref 54. Copyright 2017 Royal Society of Chemistry. (f) Schematic diagram of the reaction mechanism with LHP QDs after alcohols are added. Pathway I represents the addition of weakly polar alcohols, and pathway II represents the addition of highly polar alcohols. An arrow was added to indicate surface defects. (g) TEM image of CsPbBr_3_ suspended in hexanes. (h) TEM images of CsPbBr_3_ nanocrystals with the addition of DMF. Panels f–h are reproduced from ref 57. Copyright 2021 Royal Society of Chemistry. Distributed under Creative Commons Attribution License 3.0 (CC BY). (i–k) Photoinduced ligand detachment and morphology change of CsPbI_3_ QDs. Reproduced with permission from ref 52. Copyright 2018 American Chemical Society.

### 2.2 Synthesis Using Cationic Ligands

While polar antisolvents can postsynthetically etch perovskite QDs, or induce uncontrolled growth, the identity of the ligands used during synthesis can also influence the facets exposed on the QDs’ surface and their resulting morphology. LHP QDs synthesized by hot-injection methods have been widely observed to form nanocubes preferentially. This has been attributed to the widespread use of long-chain aliphatic oleic acid/oleylamine ligands.^45^ During the synthesis, oleate anions and oleylammonium cations are produced in situ through a weak acid–base reaction, enabling the solubilization of the necessary precursor salts. Notably, alkylammonium cations have been shown to preferentially bind to the (100) and (200) facets of LHPs, acting as a substitute for A-site cations, while carboxylates have been shown to favor replacement of the X-site anions, stabilizing the six facets that form a nanocube.^58–60^ Almeida et al. demonstrated that increasing the oleylammonium/Cs ratio during synthesis leads to a transition from nanocubes to nanoplatelets.^61^ Additionally, they observed that higher overall concentrations of oleic acid/oleylamine lead to the formation of Cs_4_PbBr_6_. Furthermore, using the ligand-assisted reprecipitation method (LARP), Sun et al. found that different combinations of long-chain acids and bases can change the shape of CsPbX_3_ QDs (Figure 3a).^62^ This shape control is linked to the micellar transition theory, where the hydrocarbon tails of the ligands determine the size and shape of the micelles. Nonetheless, such shape control, relying on long-chain acid/base ligands, still results in (100) facets being exposed on the final nanostructures, which are often anisotropic.

**Figure 3:**
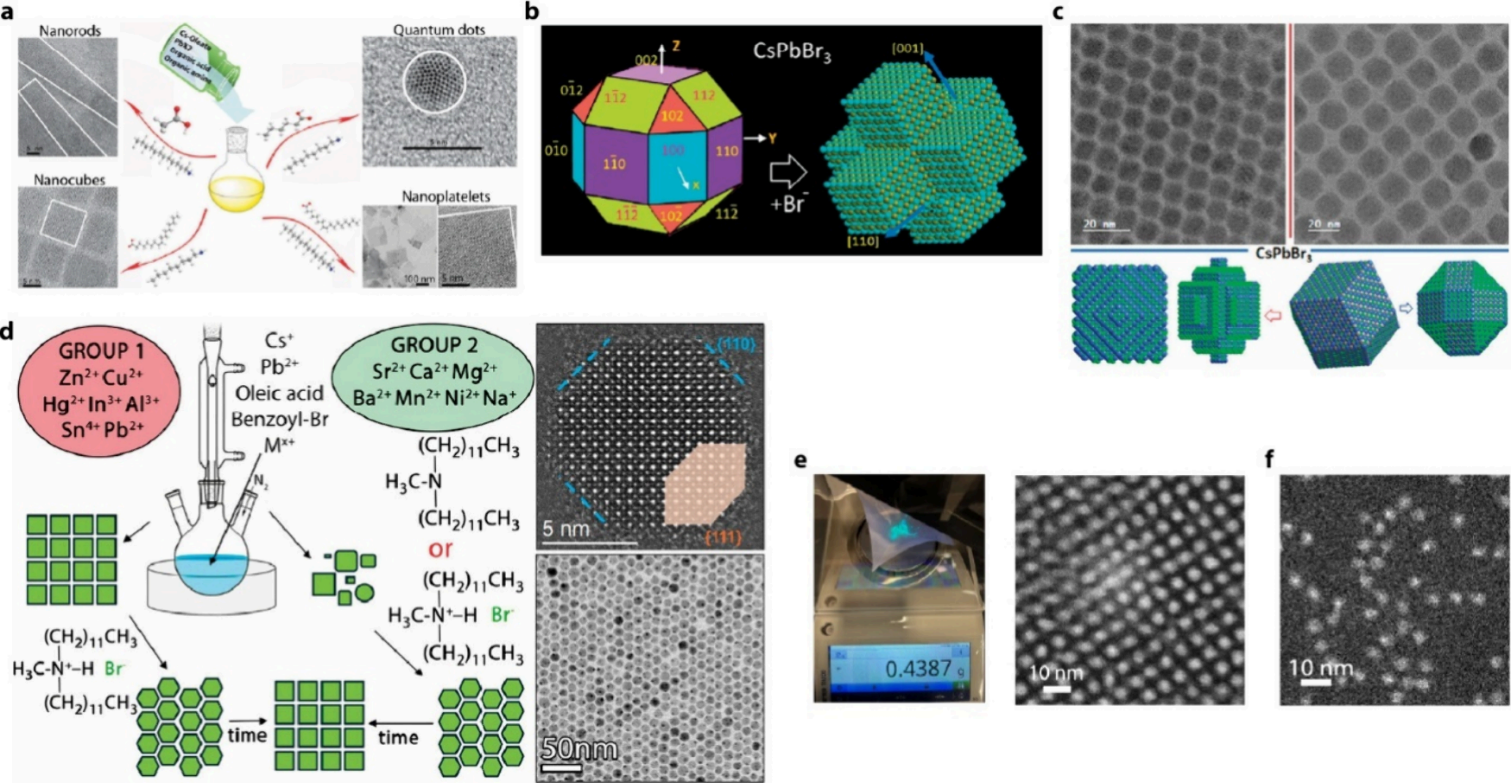
(a) Schematic illustration of different morphologies of CsPbBr_3_ nanocrystals being produced via LARP. Reproduced with permission from ref 62. Copyright 2016 American Chemical Society. (b) Schematic illustration of the transformation of polyhedral CsPbBr_3_ nanocrystals into a hexapodal-shaped nanocrystal. Reproduced with permission from ref 63. Copyright 2019 American Chemical Society. (c) Schematic illustration of nanocubes being transformed into 12-sided rhombic dodecahedron-shaped CsPbBr_3_ nanocrystals and 26-sided rhombicuboctahedron when phenylacyl bromide is used as a halide precursor. Reproduced with permission from ref 65. Copyright 2020 American Chemical Society. (d) Schematic representation of the morphology transformation of CsPbBr_3_ synthesized with additional metal cations and subsequent transformations upon the addition of didodecylmethylamine or didodecylmethylammonium bromide and subsequent annealing. Reproduced from ref 66. Copyright 2024 American Chemical Society. Distributed under a Creative Commons Attribution License 4.0 (CC-BY-NC-ND). (e) Image of 4 nm CsPbBr_3_ QD powder, with corresponding STEM image of corresponding QDs synthesized utilizing a thermodynamic equilibrium-controlled synthesis. Reproduced with permission from ref 44. Copyright 2024 American Chemical Society. (f) HAADF-STEM image of Mn^2+^-doped CsPbBr_3_ synthesized utilizing a thermodynamic equilibrium-controlled synthesis approach. Reproduced from ref 68. Copyright 2025 American Chemical Society. Distributed under a Creative Commons Attribution License 4.0 (CC-BY).

Shape or facet modulation of isotropic QDs has gained increasing interest. In 2019, Peng et al. demonstrated thermally controlled facet growth on LHP QDs.^63^ In their synthesis, seed clusters of CsPbX_3_ nanostructures were injected at high temperatures (>200 °C) and subsequently treated with alkylammonium halides. The resulting QDs feature an arm nanostructure with (001) and (110) facets exposed on the surface (in the orthorhombic phase), as shown in Figure 3b. Such controlled facet growth is attributed to the dissolution of other facets in the halide-rich environment. Upon heating, subsequent growth from monomers generated by facet dissolution along the exposed facets results in the formation of armed hexapod-shaped nanocrystals.^63^ Similar hexapod-shaped nanocrystals were later reported to be directly synthetically accessible via an open-air hot injection synthesis.^64^

Unlike primary ammonium halides, tertiary ammonium halide ligands can stabilize non-(100)-like facets on LHP QDs. It has been demonstrated that when using the α-halo ketone phenylacyl bromide and in the presence of an amine, a tertiary ammonium bromide species is generated in situ during synthesis and leads to 12-faced rhombic dodecahedron-shaped CsPbBr_3_ QDs as shown in Figure 3c.^65^ When cesium oleate is injected into the reaction mixture, it results in the formation of a mixture of cubes and hexagonal nanoplatelets, with growth primarily regulated by the oleylammonium cations present in solution. The tertiary ammonium generated over time, which preferentially passivates (200), (020), and (112) facets (in the orthorhombic phase). The multifaceted QDs are monodisperse without compromising the photoluminescence quantum yield (PLQY). Further annealing of these particles resulted in the generation of 26-sided rhombicuboctahedron nanocrystals, also shown in Figure 6d.^106^

Similarly, the protonation of the nonprimary alkylamines has been shown to regulate the shape of LHP QDs. In a recent work, Li et al. introduced a tertiary ammonium bromide or a tertiary amine into the reaction with exogenous metal cations during LHP QD synthesis.^66^ They used didodecylmethylammonium cations (DDMA^+^) or didodecylymethylamine as an etching agent to reshape CsPbBr_3_ QDs and expose (110) and (111) facets. It was discovered that the metal cations that formed strong complexes with oleates would produce cubic QDs passivated by oleates. On the other hand, cations that formed weak complexes with oleates, together with a tertiary amine, would yield QDs with a truncated cubic shape and expose multiple facets, as seen in Figure 3d. In both cases, these multifaceted QDs could be further annealed, resulting in the reformation of nanocubes.

Synthetic control of perovskite QDs can also be achieved through thermodynamic methods. In 2018, Son et al. demonstrated that the halide equilibrium between LHP QDs and the solution medium can be used to control the QD size.^40^ In this synthesis, a large amount of halide salt is added to increase the chemical potential of halides in the solution. Strongly size-confined QDs can be produced by increasing the halide concentration or lowering the reaction temperature. Furthermore, the resulting QDs are nearly free of heterogeneous broadening and exhibit a uniform cubical shape. Recently, we revealed the nanocluster-mediated QD growth mechanism under thermodynamic equilibrium control.^44^ These QDs can survive annealing at elevated temperatures without deviating from the isotropic cuboidal shape in a solution containing high concentrations of oleylammmonium bromide (Figure 3e). It is worth noting that oblate LHP QDs have also been obtained from a similar synthesis.^67^ Most recently, under thermodynamic control conditions with additional acid, spherical-shaped Mn-doped CsPbBr_3_ have been successfully synthesized with decent size uniformity (Figure 3f).^68^ The thermodynamically controlled synthesis offers a unique route to produce LHP QDs with uniform size and shape, with simultaneous high PLQY enabled by the AX termination.

### 2.3 Synthesis Using Anionic Ligands

The advances mentioned above generally involve facet etching or transformation by cationic ligands. In contrast, shape-controlled direct QD growth has been a tough question for traditional semiconductor QDs. Classic theory, such as the Wulff facet argument or Gibbs–Curie–Wulff theorem, suggests that the relative facet surface energy determines the shape of a crystal.^69^ Although it is established that high monomer concentrations can overcome thermodynamically driven facet evolution,^70^ facet-specific ligand passivation remains critical for controlling QD shape/morphology evolution. This section reviews successes in using ligands that exhibit facet-selective binding to LHP QDs.

Phosphonic acid-based ligands were proposed as an alternative ligand to the traditionally used oleic acid/oleylamine ligand pair, due to the strong binding affinity between phosphonates and Pb^2+^ ions contained on the surface of PbSe QDs.^73^ A report by Sun et al. utilized a ligand system composed of trioctylphosphine oxide (TOPO) and octylphosphonic acid to directly produce ∼10.8-nm-sized “spherical” LHP QDs, notable for being one of the pioneering reports to demonstrate a shape of CsPbBr_3_ that deviated from the typically observed “nanocube” morphology (Figure 4a).^71^ Subsequently, the Manna group studied the effects of using phosphonic acid ligands of varying chain lengths on the size and shape of CsPbBr_3_ QDs. In their report, LHP QDs were passivated with 14-carbon-chain tetradecylphosphonic acid ligands or with a mixture of octadecylphosphonic acid and a shorter-chain phosphonic acid ligands (Figure 4b). Furthermore, the QD size was tunable by modulating the combination of different phosphonic acids and octadecylphosphonic acids in a mixed-ligand synthesis, as shown in the transmission electron microscopy (TEM) images in (Figure 4c). The resulting QDs exposed Pb-terminated facets, namely, the (110) and (111) facets, in addition to the typically exposed (100) facet family.^72^ Additional works by the Manna group demonstrated that phosphonic acids could yield highly confined CsPbBr_3_ while maintaining the truncated octahedral morphology by utilizing oleylphosphonic acid. They demonstrated that oleylphosphonic acid enables complete solubilization of the precursor salts at lower temperatures (Figure 4f) than previously used, enabling lower-temperature synthetic conditions and the synthesis of highly confined truncated octahedral QDs, as seen in the TEM images in (Figure 4g).^43^ It is also worth noting that the PLQY of the multifaceted QDs synthesized following these routes is very high (close to unity), further supporting the strong binding of phosphonates on Pb^2+^-terminated facets.

**Figure 4:**
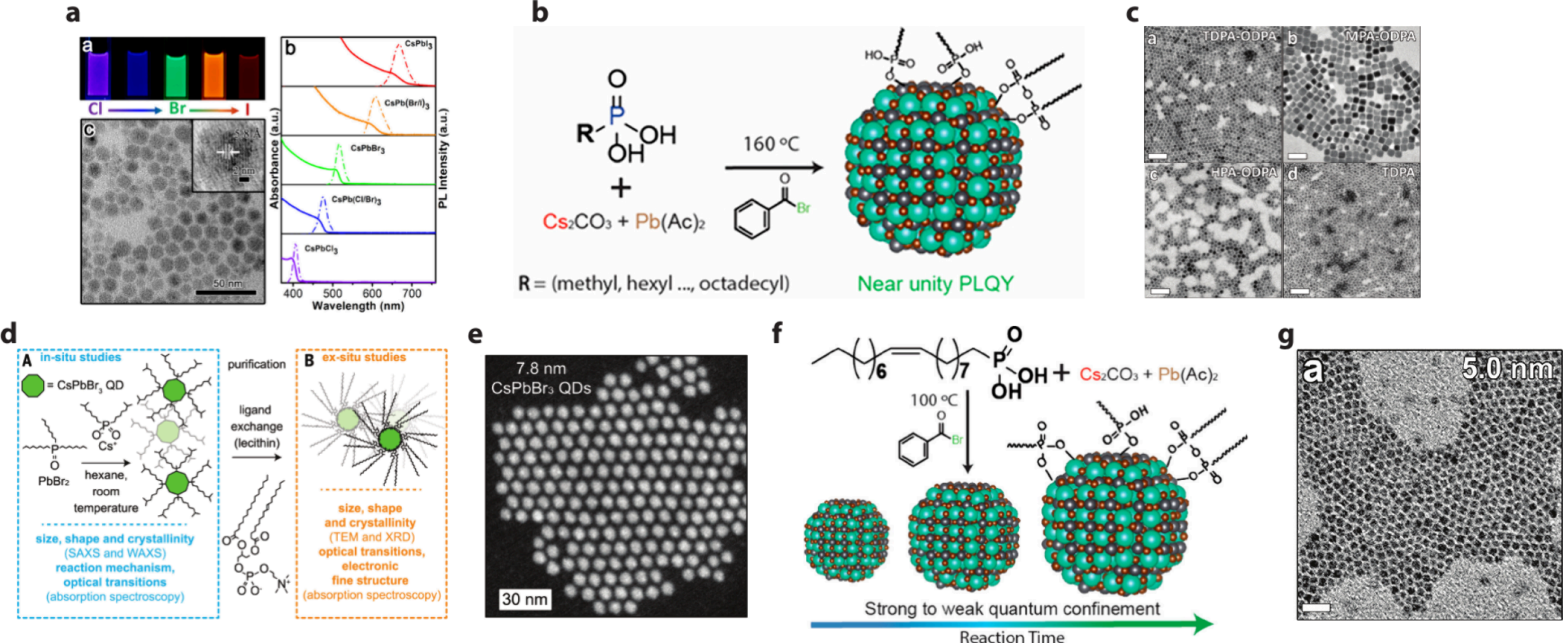
(a) CsPbX_3_ (X = Cl, Br, I) QDs synthesized utilizing octylphosphonic acid as a ligand, fluorescence images of the octylphosphonic acid capped CsPbX_3_ QDs excited under 365 nm UV lamp, photoluminescence and UV–vis absorption of the octylphosphonic acid CsPbX_3_ QDs colloids with different varying halide compositions, and a TEM image of octylphosphonic acid-capped CsPbBr_3_ QDs displaying a truncated shape. Reproduced with permission from ref 71. Copyright 2022 American Chemical Society. (b) Schematic illustration of synthesis of LHP QDs utilizing alkylphosphonic acids (c) TEM images of CsPbBr_3_ QDs prepared using different loading ratios of different alkylphosphonic acids with varying chain lengths: tetradecylphosphonic acid and octadecyl phosphonic acid (ratio 3:1), methylphosphonic acid and octadecylphosphonic acid (ratio 1:3), hexylphosphonic acid and octadecylphosphonic acid (ratio 1:3), and exclusively tetradecylphosphonic acid. Reproduced with permission from ref 72. Copyright 2019 American Chemical Society. (d) Reaction scheme and overview of in situ monitoring techniques utilized to monitor the growth of LHP QDs synthesized utilizing the TOPO/DOPA synthesis. Overview of the used ex situ techniques on ligand-exchanged and washed QDs. (e) STEM image of washed 7.8 nm CsPbBr_3_ QDs showing truncation synthesized using the TOPO/DOPA route. Panels d and e are reproduced with permission from ref 41. Copyright 2022 American Association for the Advancement of Science. (f) Schematic illustration of the colloidal synthesis of CsPbBr_3_ QDs utilizing oleylphosphonic acid as a ligand. (g) TEM images of highly confined CsPbBr_3_ synthesized using oleylphosphonic acid. Reproduced from ref 43. Copyright 2020 American Chemical Society. Distributed under a Creative Commons Attribution License (CC-BY).

Phosphonic acids have also been demonstrated to control the growth of LHP QDs in room-temperature synthesis. In a report by Akkerman et al., trioctylphosphine oxide/diisoctylphosphonic acid (TOPO/DOPA) was used to regulate the nucleation and growth of LHP QDs (Figure 4d).^41^ It was demonstrated that TOPO can prevent the initial conversion of PbBr_2_ into a reactive PbBr_3_^–^ species. The conversion occurs upon the introduction of Cs-DOPA, which when a sufficient [PbBr_3_^–^] is reached, only then does nucleation of the QDs occur. This slow conversion allows continuous monomer generation postnucleation, thereby decoupling growth from the initial nucleation of the QDs. This is particularly advantageous as monodisperse ensembles 3–13 nm in size were demonstrated for both hybrid and all-inorganic perovskite QDs.^13,14^ The resulting QDs were rhombicuboctahedral, as shown in (Figure 4e), presumably due to the presence of diisoctylphosphonate, consistent with reports using other phosphonic acids. This truncated rhombicuboctahedron is also observed when utilizing additional classes of ligands, such as sulfonium-based ligands.^74^

### 2.4 Lead-Free Perovskite Nanocrystals

The advances in synthesis discussed earlier show how antisolvents and ligands can be used to control the shape of LHP nanocrystals. Despite their excellent optical properties, lead cations’ toxicity raises health concerns. Substitution of lead in the perovskite lattice has been explored to produce lead-free perovskite nanocrystals. This section discusses some recent advances in the morphology control of tin-based and double perovskite nanocrystals.

One of the widely studied lead-free perovskites is ASnX_3_ (A = Cs, FA, MA; X = Br, I). Hot-injection-based approaches have been used to produce size-tunable nanocrystal ensembles with narrow size distributions.^75–80^ It has been suggested that CsSnBr_3_ exhibits a cubic unit cell (*Pm*3*m*),^79,200^ whereas CsSnI_3_ displays an orthorhombic crystal structure.^77^ As with LHPs, the most widely observed nanocrystal morphology is cubic.^79–81^ This should not be surprising since the crystal structure and the surface passivation are akin to LHP nanocrystals. Efforts have been made on the synthesis control of cesium tin halide nanocrystals. Wang et al. demonstrated that utilizing tin 2-ethylhexanoate as a tin precursor resulted in the formation of a hollow CsSnBr_3_ nanocage at higher reaction temperatures (230 °C).^82^ It was proposed that the oriented attachment of the ethylhexanoate group drives a growth-driven self-assembly process, leading to the formation of the observed nanocage structures. Importantly, the formation of the nanocage exhibits enhanced oxygen resistance. Additionally, perovskite derivative Cs_2_SnI_6_ nanocrystals have also been synthesized with spherical morphology and anisotropic structures.^83^

The study of the optical properties of tin halide perovskite nanocrystals is hindered by the oxidation of Sn­(II) to Sn­(IV), which degrades the nanocrystals and reduces photoluminescence (PL). This remains one of the main barriers to identifying optical property changes associated with nanocrystal morphology. Synthesis methods that can improve stability and boost PL are needed to produce high-quality tin-based nanocrystals. Advances in controlling precursors and in antioxidation protocols have proven effective.^77,84^ Future understanding of the growth mechanism and the development of precise synthesis control will be essential.

Elpasolites, or double perovskites, offer another route to design lead-free perovskite nanocrystals by replacing divalent Pb cations with monovalent and trivalent Pb-free cations.^85–87^ Double perovskite nanocrystals have the general formula A_2_B^I^B^III^X_6_ and are widely reported to exhibit a cubical morphology.^85,88–93^ Nevertheless, different nanocrystal morphologies have been demonstrated. Lee et al. reported that, by adjusting the reaction temperature during a hot-injection-based synthesis of Cs_2_NaBiX_6_ nanocrystals, both cuboctahedral and cuboidal morphologies were attainable.^94^ Heating the cuboidal nanocrystals to 200 °C led to particle growth, exposing the (100) and (111) facets and a cuboctahedral shape. Interestingly, new spectroscopic features emerge in cuboctahedral nanocrystals, which were attributed to heterostructure growth on the newly exposed facets. Liu et al. demonstrated that nanocrystals of Cs_2_AgIn_*x*_Bi_1–*x*_Br_6_ with triangular, hexagonal, and cuboidal morphologies could be obtained by adjusting reaction temperature.^95^ Specifically, cuboidal particles were obtained at 150 °C, hexagonal at 170 °C, and triangular at 190 °C. It was also suggested that the passivation at elevated temperatures and the instability of Ag^+^ made cube nanocrystals more stable.

## 3 Morphology-Dependent Optical Properties of LHP QDs

The size confinement of QDs makes their photophysical properties highly sensitive to surface conditions. Specifically, the atomic arrangement, coordination environment, defect types, geometric pattern of binding sites, ligand binding strength, and surface polarity can vary significantly with exposed facets and their surface passivation, affecting exciton and multiexciton dynamics in QDs. Additionally, the shape of the QDs, which depends on their surface facet exposure, can influence the symmetry and boundary conditions of the electric field within them. This section reviews the latest advances and understanding of how facet-dependent physical and optical property changes occur in isotropic, zero-dimensional (0D) LHP QDs.

### 3.1 Exciton Degeneracy

For QDs possessing a nonspherical shape, a reduction of the symmetry can lead to splitting of the band-edge exciton states.^96,97^ By investigating absorption spectra of CsPbBr_3_ QDs, Akkerman et al. found that absorption peaks are more well-resolved in spheroidal QDs compared to cubical QDs, even with similar size dispersion (Figure 5a,b).^41^ Calculations using the effective-mass approximation indicate that cubic symmetry mixes higher-order exciton states, as shown in Figure 5c,d. This also results in a slight splitting of higher-energy absorption states, smoothing the spectra and broadening the absorption peaks (Figure 5b). This spectroscopic feature can serve as a guide for synthesizing perovskite QDs, offering a quick assessment of QD shape via simple absorption measurements.

**Figure 5:**
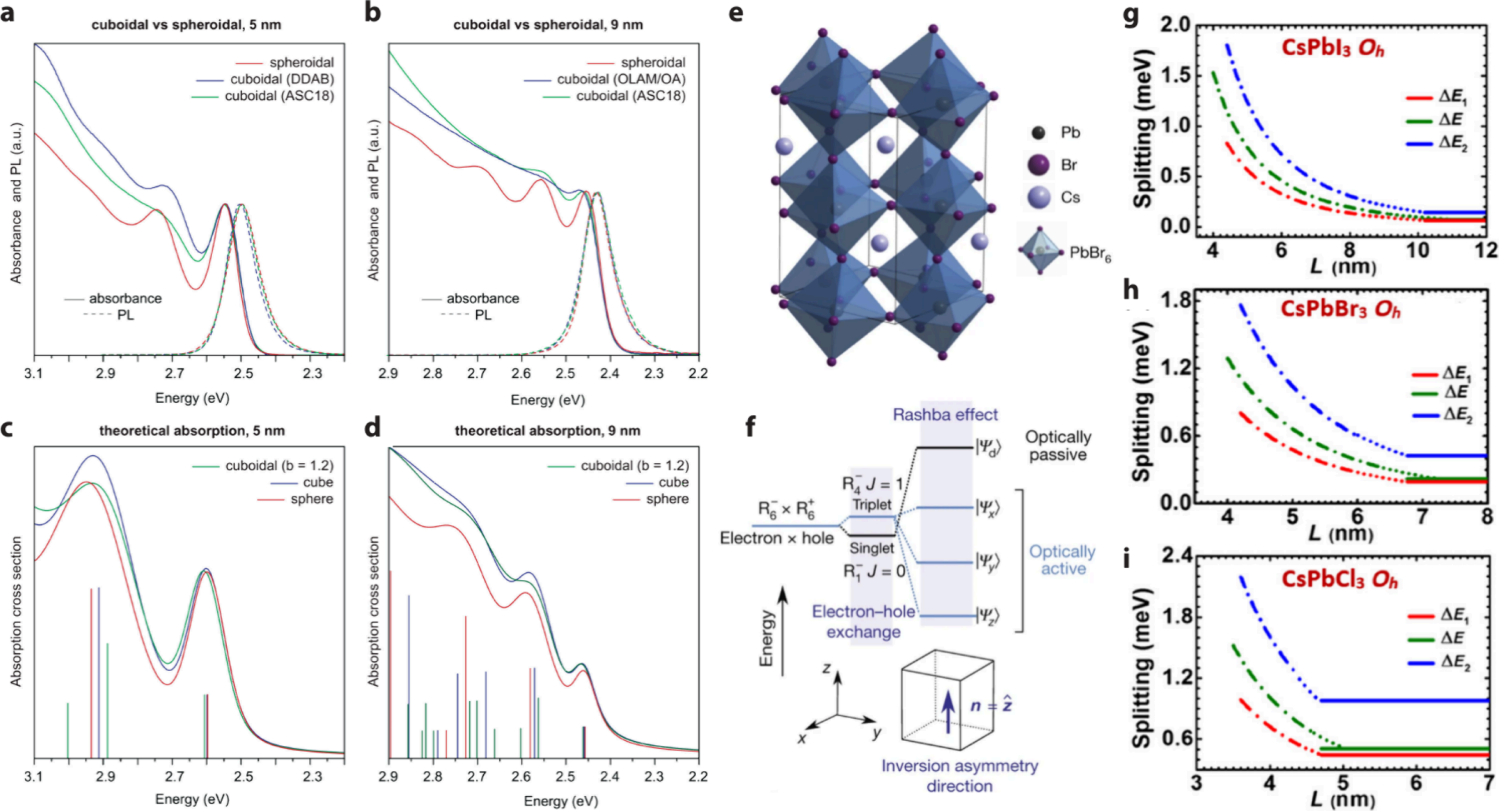
(a and b) Absorption and PL spectra of cuboidal and spherical CsPbBr_3_ perovskite QDs with sizes of 5 and 9 nm, respectively. (c and d) Calculated absorption cross sections of the QDs corresponding to panels a and b. Panels a–d are reproduced with permission from ref 41. Copyright 2022 American Association for the Advancement of Science. (e) Orthorhombic crystal structure of CsPbBr_3_ (*orthorhombic*, unit cell shown as a frame), which deviates from the ideal cubic perovskite structure by an octahedral tilting. (f) Predicted excitonic fine structure considering short-range electron–hole exchange (middle) and then including the Rashba effect (right) under orthorhombic symmetry for CsPbBr_3_ (unit cell shown in the bottom). Panels e and f are reproduced with permission from ref 98. Copyright 2018 Springer Nature. (g–i) Computed CsPbX_3_ perovskite QD exciton fine structure splitting with respect to their size, by assuming a cubic (*O*_*h*_ symmetry) lattice and an anisotropic cuboidal geometry with an aspect ratio of 0.9:1:1.1. The lifted degeneracy is due to the shape anisotropy since the lattice is isotropic. Panels g–i are reproduced with permission from ref 99. Copyright 2019 American Physical Society.

Exciton fine structure splitting in LHP QDs is also related to their shape. The cubical shape of typical LHP QDs results in an inhomogeneous electric-field distribution of a photon. Such a shape-dependent fine-structure splitting has been initially predicted theoretically. Becker et al. proposed that the electron–hole exchange interaction, together with the Rashba effect, has led to a bright triplet ground state (Figure 5e,f).^98^ Lounis and colleagues then presented experimental evidence that the dark exciton remains the ground state in FAPbBr_3_ nanocrystals.^98,100,101^ Nevertheless, the triplet bright exciton state can be split by the anisotropy of the unit cell (Figure 5f). Meanwhile, Ben Aich et al. have addressed the often-overlooked impact of QD shape anisotropy on the excitation fine-structure splitting.^99^ Using group-theoretical arguments and the ***k*** · ***p*** approximation, they found that the cuboidal shape anisotropy also introduces fine-structure splitting, even when a highly symmetric cubic lattice is assumed (Figure 5g–i). Fine structure splitting of LHP QDs has been experimentally verified by single-particle spectroscopy. PL spectra of single CsPbBr_3_ QDs showed three peaks with near-linear polarizations at cryogenic temperatures with subns decay lifetimes.^100,102,103^

### 3.2 Shape-Related Exciton Dynamics

QDs with different shapes expose different facets, which can affect their blinking behavior under different surface-defect passivation conditions. Mi et al. found that blinking in single CsPbBr_3_ QDs can be nearly eliminated by epitaxial ligand molecular crystal growth on the (100) facet. Such a crystal matrix also enabled extraordinary single-photon emission photostability (Figure 6a).^26^ This strategy relies on the proximity of neighboring surface binding sites (Cs^+^ vacancies) because the ligands pack via attractive π–π interactions, which fade quickly with distance. In contrast, polyhedral QDs, which expose (110) and (111) facets, have binding sites that are farther apart. The ligand crystallization is compromised, leading to single-QD PL intensity fluctuations at high excitation intensities (Figure 6b).^104^ It is also worth noting that *g* ^(2)^(0) value may not accurately represent the biexciton emission yield if LHP QDs exhibit intense blinking. We have recently experimentally demonstrated a nearly 60% overestimation of CsPbBr_3_ QDs’ biexciton emission yield when blinking (Figure 6c).^105^

**Figure 6:**
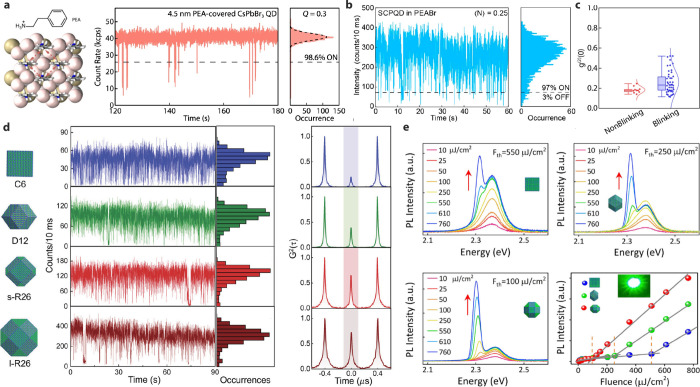
(a and b) Blinking traces of cubical (a) and polyhedral (b) CsPbBr_3_ QDs. The scheme illustrates the epitaxial QD-ligand molecular crystal interface. Panel a is reproduced from refs 26 and 104. Copyright 2025 Springer Nature. Distributed under Creative Commons Attribution License 4.0 (NC-ND-BY). Panel b is reproduced with permission from ref 104. Copyright 2023 American Chemical Society. (c) Statistics of the *g* ^(2)^(0) values of blinking and nonblinking LHP QDs with similar sizes. Reproduced with permission from ref 105. Copyright 2025 Springer Nature. (d) Schematic of cube (C6), rhombic dodecahedron (D12), small-sized rhombicuboctahedron (s-R26), and large-sized rhombicuboctahedron (l-R26) shaped QDs and their representative blinking traces and PL intensity distributions. Second-order photon correlation function (antibunching) of the corresponding QDs. Reproduced with permission from ref 106. Copyright 2024 American Chemical Society. (e) ASE for facet-engineered CsPbBr_3_ nanocrystals. The PL spectra for cubic, rhombic dodecahedron, and rhombicuboctahedron nanocrystals are represented as a function of excitation fluence. The PL intensity of these nanostructures is also represented as a function of excitation fluence, where the dashed vertical lines indicate the onset of ASE. Reproduced with permission from ref 107. Copyright 2022 American Chemical Society.

Different surface facets exhibit distinct atomic compositions, leading to different polarities due to charge imbalance. Such surface polarity can also affect multiexciton interactions in QDs. Titus et al. synthesized CsPbBr_3_ QDs at a similar volume but with 6-, 12-, and 26-faceted polyhedral shapes (top three panels in Figure 6d). By measuring the second-order photon correlation (*g* ^(2)^) function, they found that the biexciton PLQY of 12- and 26-faceted QDs nearly doubled compared to the 6-faceted cubic QDs (Figure 6d). This suggests that additional facets lead to a lower biexciton Auger recombination rate.^106^ The reduced Auger recombination rate in multifaceted QDs is attributed to a decrease in exciton binding energy due to increased dielectric screening as surface polarity increases when more polar facets are exposed. In their atomic model, the 6-faceted cubic QDs possess an orthorhombic phase, exposing four (110) facets and two (200) facets. The 12-faceted rhombic dodecahedron-shaped QDs have (200), (020), and (112) facets that exhibit higher polarity due to the separation of cation- and anion-dominated planes along the perpendicular directions of these facets. Similarly, the 26-faceted rhombicuboctahedron QDs also include more polar (101) facets. In addition to morphological effects, increasing the QD volume will also affect multibody interactions, further reducing the Auger rate (bottom panel of Figure 6d). Consequently, the 26-faceted QDs showed an average *g* ^(2)^(0) of 0.4, significantly higher than the 0.21 observed in the cubic QDs.

### 3.3 Shape-Related ASE/Lasing in Perovskite QDs

This suppression of the Auger rate in polyhedral nanocrystals has also been applied to CsPbX_3_ nanocrystals to investigate their potential as optical gain media. A report by Bera et al. shows that facet engineering promotes the amplified spontaneous emission (ASE) from LHP QDs. In this study, three different shapes of CsPbBr_3_ were tested: cube, rhombic dodecahedron, and rhombicuboctahedron, to examine how shape affects the ASE threshold (Figure 6e).^107^ Increasing the number of facets from 6 (cube) to 12 (rhombic dodecahedron) lowered the gain threshold by a factor of 2.2. This is due to the low nonradiative Auger recombination rates and quick thermalization to the emitting states. A similar trend was seen with the 26-faceted rhombicuboctahedron, which achieved ASE gain at a much lower threshold, further confirming that facet engineering reduces the gain threshold (Figure 6e).

Implementing perovskite QDs as a lasing gain material remains challenging due to their relatively fast Auger recombination rates and limited photostability, especially when intense excitations are required to achieve the optical gain. Due to the efficient two-photon absorption of perovskite, successful upconversion perovskite QD ASE/lasing has been achieved.^108–110^ Additionally, the excellent nonlinear saturable absorption of CsPbBr_3_ nanocrystals in the C-band has enabled a Q-switched fiber laser at 1560 nm.^111^ Recently, ultrastable colloidal perovskite nanocrystal laser has been demonstrated.^112^ In this report, the laser’s efficiency is still limited by the relatively short gain lifetime, which can, however, be enhanced by the double-pump excitation scheme. Although most perovskite ASE/lasing is demonstrated primarily in cubic QDs, they still suggest that biexcitons are crucial for efficient lasing. This leaves ample room to explore the impact of surface-facet engineering of perovskite nanocrystals on multicharge-carrier engineering and on reducing the ASE/lasing threshold.

## 4 Effect of QD Morphology on the Photocatalytic Properties and Heterostructures

In this section, we will explore how the shape and surface facet exposure of LHP QDs affect photocatalysis and QD heterostructures. The typical cubic-shaped CsPbX_3_ QDs demonstrate excellent optical properties, with PLQYs reaching up to 100%. Although highly emissive, these cubic QDs show relatively low catalytic activity due to rapid radiative recombination of charge carriers. In contrast, the noncubic CsPbX_3_ QDs with low PLQY (<1%) display improved catalytic activity. The exposed facets provide pathways for charge-carrier transport via surface states, surface adsorption, and chemical modifications, facilitating charge separation and suppressing fast radiative recombination, thereby enabling charge-carrier transfer to an acceptor and favoring applications such as photocatalysis, charge transfer, and photoconductivity. The lattice-matching condition of exposed facets also influences the material design for QD heterostructure growth.

### 4.1 Photocatalytic Properties

Shyamal et al. demonstrated the effect of exposed facets by analyzing the photocatalytic activity of noncubic CsPbBr_3_ structures.^113^ The CO_2_ reduction rate served as a measure of the catalytic performance of these nanostructures. In this study, three different shapes of CsPbBr_3_—polyhedral noncubes, hexapods, and cubes—were synthesized, each with distinct exposed facets (Figure 7a). The multifaceted polyhedral QDs were found to be a better catalyst than the others (Figure 7b). This illustrates how the greater number of exposed facets, combined with lower emissivity, results in improved catalytic performance.^113^

**Figure 7:**
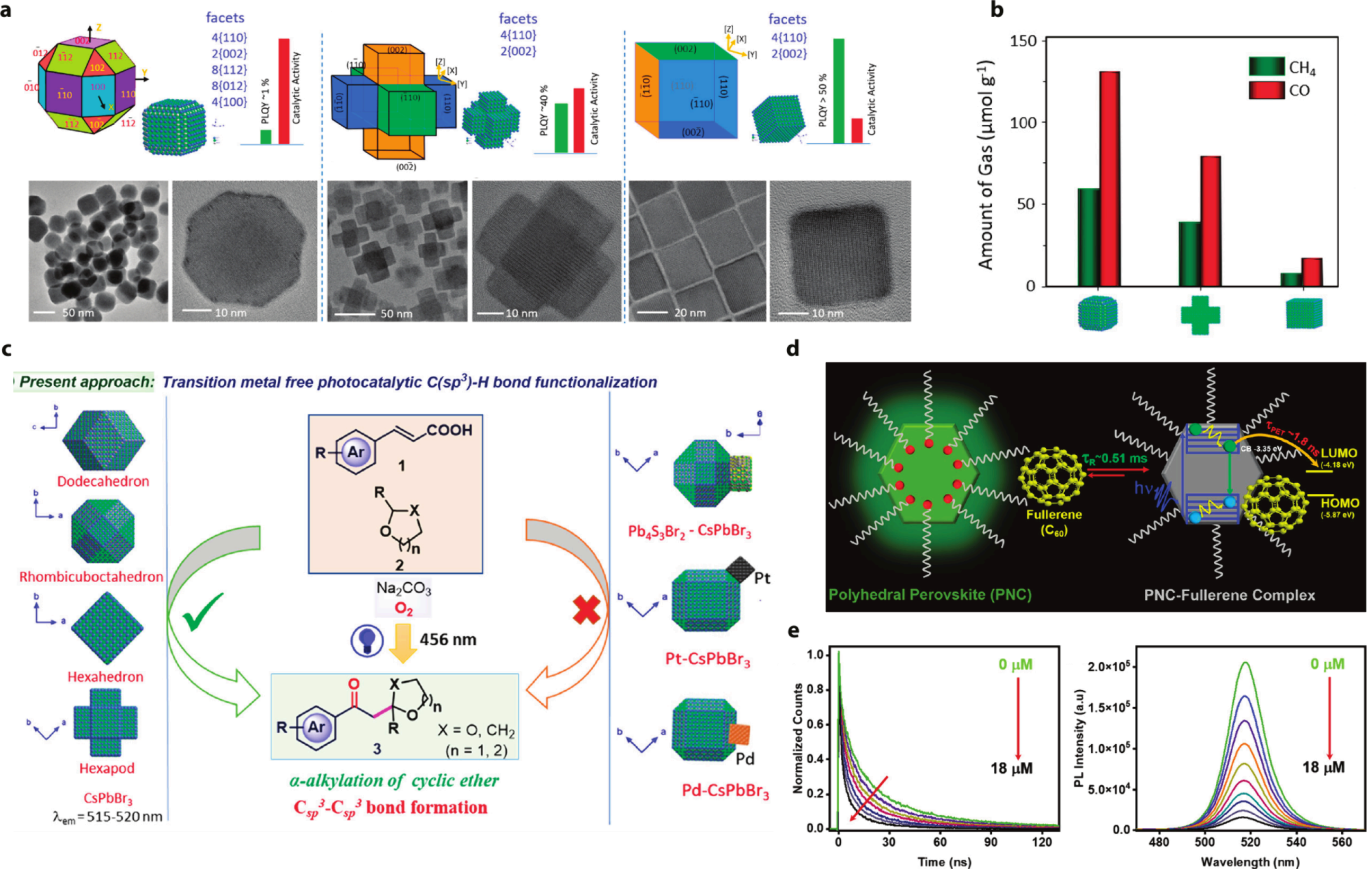
Applications of the morphologically controlled CsPbX_3_ nanocrystals. (a) Models showing polyhedron-shaped noncubes, hexapods, and cubic CsPbBr_3_ nanostructures, along with the TEM and high-resolution TEM images for each of the structures. The models also demonstrate the facets exposed on each nanostructure. (b) Histograms showing the formation of CO and CH_4_ from CO_2_ reduction after 4 h using polyhedron, hexapod, and cubic CsPbBr_3_ nanostructures. Panels a and b are reproduced with permission from ref 113. Copyright 2020 American Chemical Society. (c) Schematic illustration of the photocatalytic reaction pathways for α-alkylation of tetrahydrofuran in the presence of different-shaped CsPbBr_3_ photocatalysts. Reproduced with permission from ref 114. Copyright 2024 American Chemical Society. (d) Illustration of complexation reaction of polyhedral CsPbBr_3_ nanocrystal with fullerene, facilitating PET. (e) PL transients and PL spectra of the nanocrystals dispersed in toluene at different fullerene concentrations. Panels d and e are reproduced with permission from ref 115. Copyright 2023 American Chemical Society.

Other than CO_2_ reduction, polyhedral CsPbBr_3_ nanostructures have also been used for room temperature C–H bond activation. Mondal et al. demonstrated the functionalization of the C_sp_^3^–H bond using CsPbBr_3_ nanostructures having different exposed facets (Figure 7c).^114^ The overall results suggested that the dodecahedron-shaped CsPbBr_3_ nanocrystals having (112), (200), and (020) facets (orthorhombic) were the most efficient for C–H bond functionalization due to higher surface adsorption of the reactants, producing turnover numbers as high as 32200.

Further demonstrations have shown that polyhedral CsPbBr_3_ nanocrystals can be used for charge-transfer studies, enabling comparisons with their cubic counterparts. Fullerene C_60_ was complexed with 12-faceted dodecahedral CsPbBr_3_ nanocrystals, and the photoinduced electron transfer (PET) was studied from the nanocrystal to the fullerene. The increased number of exposed facets provides more binding sites for the fullerene to form complexes with the nanocrystals and to scavenge photogenerated electrons from the conduction band. Their study suggests that PET is more efficient when attached a fullerene to the nanocrystal, highlighting the potential of surface-engineered nanocrystals for charge-transfer applications (Figure 7d,e).^115^

### 4.2 LHP QD Heterostructure

CsPbX_3_ QD-based photodetectors are of significant interest due to their high absorptivity, scalable synthesis, and solution processability. However, the sizeconfinement in 0D CsPbX_3_ QDs may lead to rapid charge-carrier recombination, a major energy-loss pathway in photocatalytic reactions and single-junction photovoltaic devices. Charge separation is therefore crucial for enhancing the photosensitivity of a device made with CsPbX_3_ QDs, and an effective strategy to achieve this is to incorporate a heterointerface with another semiconductor on the perovskite surface, which results in type-II band-edge alignment. Constructing heterostructures on LHP QDs has recently attracted attention.

To achieve epitaxial growth, lattice matching is required between the surface facet exposed on LHP QDs and the other crystal. Rajdeep et al. explored the epitaxial growth of Pb_4_S_3_Br_2_ on various shapes of CsPbBr_3_ and their photocatalytic activity for CO_2_ reduction (Figure 8a,b).^116^ The polyhedral nanostructure exhibited ideal facet connections due to lattice matching. In these QDs, both (200) and (110) facets (orthorhombic) are epitaxially connected with lead sulfobromide. The cubic nanocrystals, on the other hand, did not form heterostructures, further indicating the effect of facet-selective epitaxial growth. The heterostructures exhibit type-II band alignment, thereby significantly reducing the PLQY (Figure 8c) but enhancing the catalytic activity of CsPbBr_3_.^116^

**Figure 8:**
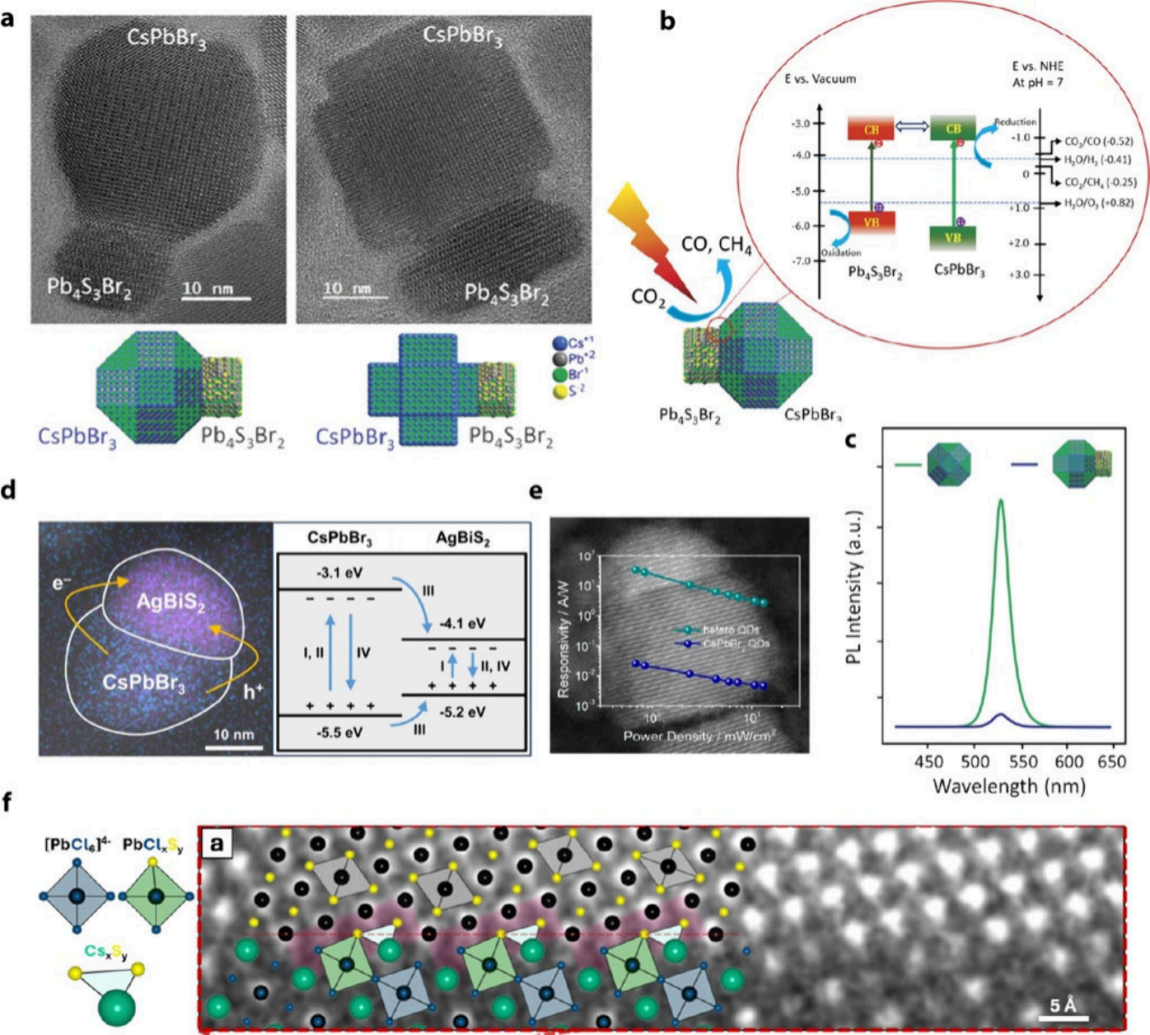
(a) High-resolution TEM of rhombicuboctahedron and hexapod-shaped CsPbBr_3_–Pb_4_S_3_Br_2_ heterostructures along with the atomic models. (b) Schematic presentation of the energy band diagram and photocatalytic CO_2_ reduction at the junction of the poly(110) CsPbBr_3_–Pb_4_S_3_Br_2_ heterostructure. (c) PL spectra of rhombicuboctahedron CsPbBr_3_ nanocrystals and poly(110) CsPbBr_3_–Pb_4_S_3_Br_2_ heterostructure. Panels a–c are reproduced with permission from ref 116. Copyright 2022 American Chemical Society. (d) CsPbBr_3_–AgBiS_2_ Janus heteronanocrystal shown in TEM using energy-dispersive spectroscopy mapping along with a schematic representation of the quasi-type-II band alignment. Reproduced with permission from ref 117. Copyright 2025 American Chemical Society. (e) CsPbBr_3_–Pb_4_S_3_Br_2_ Janus heterostructure shown using HAADF STEM, along with the responsivity of two photoconductor devices as a function of the power density. Reproduced with permission from ref 118. Copyright 2023 American Chemical Society. (f) HAADF-STEM image of the CsPbCl_3_–PbS stair-like epitaxial interface. Reproduced from ref 119. Copyright 2024 American Chemical Society, distributed under a Creative Commons 4.0 Attribution License.

Significant work has gone into developing different epitaxial perovskite heterostructures featuring a wide variety of material systems, such as the CsPbX_3_–lead chalcohalides,^120^ metal perovskite (Au–CsPbBr_3_),^121^ metal chalcogenide–perovskite (PbS–CsPbX_3_,^122^ ZnS–CsPbX_3_ ^123,124^ PbSe–CsPbX_3_,^125^ and PbTe–CsPbBr_3_), Cs_4_PbBr_6_–CsPbBr_3_,^126^ CsPbBr_3_–Bi_2_PbS_4_,^127^ and AgBr–CsPbBr_3_.^128^ Recent studies have shown that Pb_4_S_3_X_2_ has a (101) perovskite facet-like atomic plane, and CsPbX_3_–Pb_4_S_3_X_2_ heterostructures have been successfully synthesized using epitaxial growth, along the (101), (010), and (001) facets of CsPbBr_3_, which exhibit quasi-type-II band alignment, leading to almost complete emission quenching from the perovskite counterpart.^120,129^ Employing this concept, Zhang et al. developed a high-performance photoconductor based on CsPbBr_3_–Pb_4_S_3_Br_2_ nanocrystal heterostructures.^118^ These nanocrystals are Janus-shaped, with Pb_4_S_3_Br_2_ epitaxially growing on the (101) facet (orthorhombic) of the cubic perovskite structure (Figure 8d). The quenched PL intensity of the Janus nanocrystals at 520 nm indicated a reduction in the radiative recombination process of the CsPbBr_3_ domain. The energy-transfer efficiency, estimated by comparing the PLQY of the heterostructure to the pristine CsPbBr_3_, was above 95%. The efficient charge transfer resulting from the quasi-type-II band alignment led to outstanding device performance, including a responsivity of 34.0 A W^–1^, a light-to-dark current ratio of 1.1 × 10^5^, and a specific detectivity of 1.26 × 10^14^ Jones.

Qiu et al. demonstrated the performance of CsPbX_3_–AgBiS_2_ Janus heterostructure nanocrystal photoconductor.^117^ In this heterostructure, the interface was formed on the (200) facet of the CsPbBr_3_ counterpart. The efficient charge transfer and high absorptivity produced impressive photoconductor responsivity (183.8 A W^–1^) and specific detectivity (5.0 × 10^14^ Jones). Thus, the importance of facet-dependent epitaxial growth of other semiconductors on CsPbX_3_ nanocrystals is highlighted through these reports. Efficient charge-carrier transfer through nanoheterojunctions has significant potential for optoelectronic applications.

While many studies on heterostructure growth focus on facets with a (100) plane-like (cubic) atom arrangement and a type-II band alignment, a recent report by Livakas et al. demonstrated metal sulfide heterostructures on CsPbCl_3_ nanocrystals on (−210) planes.^119^ In this work, the lattice-matching condition was closely investigated using high-resolution scanning transmission electron microscopy (STEM) imaging and DFT. Figure 8f shows the interface of the CsPbCl_3_–PbS epitaxial heterostructure. The stair-like alignment enables full coordination of Pb^2+^ cations without halide vacancies. Such a structure enables strong luminescence from PbS and charge-carrier funnelling from CsPbCl_3_ due to the type-I band alignment. They further successfully synthesized CsPbBr_3_–PbS and CsPbCl_3_–Cu_2–*x*_S through anion and cation exchanges using the CsPbCl_3_–PbS heterostructure.

## Conclusion and Outlooks

Since the emergence of LHPs as a new family of colloidal QDs, significant progress has been made in harnessing their unique properties for various applications. Compared to conventional QDs, better control over synthesis and a better understanding of the structural-property relationship of LHP QDs still require further effort. This review discusses recent developments in the emerging field of controlling the shape of LHP QDs across three areas: synthesis, optical properties, and applications.

Although progress has been made in controlling the facet exposures of LHP QDs, most synthetic methods still have limited ability to tune their size and morphology. Additionally, while the uniformity of LHP size has significantly improved over the past decade, less attention has been given to the uniformity of shape or morphology in multifaceted LHP QDs. To date, achieving simultaneous control over size and shape in highly inhomogeneous LHP QDs has fallen short of that in conventional QDs. Currently, the understanding of both the thermodynamics and kinetics of facet growth remains incomplete. Moreover, the design of ligands for highly ionic LHP QDs capable of passivating facets other than the (100) family remains limited. Furthermore, the growth mechanisms of LHP QDs under various reaction conditions and with different ligands need further exploration to enable regulation beyond size and composition. This calls for efforts to capture intermediate products more effectively and characterize them, as well as to monitor the kinetics of QD evolution during synthesis using different routes. Additionally, the thermodynamic control protocol is promising for simultaneously achieving precise size and shape regulation, which warrants further attention.

Correlating the shape and optical properties of QDs relies on materials and spectroscopic characterizations. Characterizations using QD ensembles inevitably convolute shape and size inhomogeneities with measured spectroscopic features. While single-particle spectroscopy is free of ensemble averaging, it often lacks statistical significance and is prone to selection bias. Additionally, single-particle spectroscopy often requires significantly diluted colloids, during which LHP QDs can be subject to surface damage. Addressing these issues would require highly uniform, structurally stable LHP QDs, and continued efforts on synthesis development and surface ligand engineering are needed.

The applications of morphologically controlled LHP QDs can clearly benefit from improved synthesis control and optical research. Facet-specific passivation strategies should be considered to enhance the stability of LHP QDs with various shapes, since most advanced designer ligands target (100) facets. Additionally, the use of strongly confined LHP QDs of different shapes should be explored and incorporated into applications to harness new properties arising from quantum confinement and exciton-surface interactions. Following recent advances, the pathway to a deeper understanding of the synthesis and properties of LHP QDs will be paved by future efforts from a broader community.
